# Seasonal Variation in the Prevalence of Sand Flies Infected with *Leishmania donovani*

**DOI:** 10.1371/journal.pone.0061370

**Published:** 2013-04-09

**Authors:** Puja Tiwary, Dinesh Kumar, Mukesh Mishra, Rudra Pratap Singh, Madhukar Rai, Shyam Sundar

**Affiliations:** Infectious Disease Research Laboratory, Department of Medicine, Institute of Medical Sciences, Banaras Hindu University, Varanasi, Uttar Pradesh, India; The George Washington University Medical Center, United States of America

## Abstract

**Background:**

Visceral Leishmaniasis (VL) is a life threatening neglected infectious disease in the Indian subcontinent, transmitted by the bite of female sand flies. Estimation of the infectivity in the vector population, collected in different seasons, may be useful to better understanding the transmission dynamics of VL as well as to plan vector control measures.

**Methodology:**

We collected sand flies from highly endemic regions of Bihar state, India for one year over three seasons. The species of the sand flies were confirmed by species-specific PCR-RFLP. *Leishmania donovani* infection was investigated in 1397 female *Phlebotomus argentipes* using PCR, targeting the *Leishmania* specific minicircle of the kDNA region. Further, the parasitic load in the infected sand flies was measured using quantitative PCR.

**Conclusion:**

Though sand flies were most abundant in the rainy season, the highest rate of infection was detected in the winter season with 2.84% sand flies infected followed by the summer and rainy seasons respectively. This study can help in vector elimination programmes and to reduce disease transmission.

## Introduction

In vector borne diseases, the seasonality of the vector species directly affects the transmission of the infectious disease. Thus, the study of insect behaviour in natural settings is crucial to understanding the current status of any disease. Leishmaniases are some of the most severe forms of vector borne infectious diseases, affecting 98 countries worldwide. In India, visceral leishmaniasis (VL) is a major cause for morbidity and mortality. It is prevalent in the four states of Bihar, Jharkhand, West Bengal and Uttar Pradesh, with about 84% of all VL cases being reported from Bihar [1].

Sand flies, the vectors for leishmaniasis, belong to the genus *Phlebotomus* in the old world and *Lutzomyia* in the new world. In India, VL is caused by infection with the protozoan parasite *Leishmania donovani* with female *Phlebotomus argentipes* considered as the vector [2]. The female sand fly needs blood for its eggs to develop, and becomes infected with the *Leishmania* parasite while biting an infected individual or animal. Over a period of 6 to 9 days, the parasite develops inside the sand fly gut [3]. When an infected female sand fly feeds on an uninfected person or animal, it simultaneously inoculates the parasite, thus completing the transmission cycle. The incubation period from infection to clinical VL is between 2 to 6 months [4].

Data on the population dynamics of *P. argentipes* are scanty from the Indian subcontinent. Changes in climate affect the density and behavioural bionomics of a vector species [5], [6], [7], [8] and thus disease transmission. In India, three seasons occur throughout the year, each with its distinct temperature and humidity. Furthermore, seasonal fluctuations in the pattern of VL transmission have been reported [9] that indicate seasonal variations in rates of *L. donovani* infection in the female *P. argentipes* vector that may in turn have direct impact on VL transmission throughout the year. In this study, seasonal variations in the *P. argentipes* population, the infection rate and the parasitic load were investigated employing molecular methods.

## Materials and Methods

### Study area

**Figure 1:**
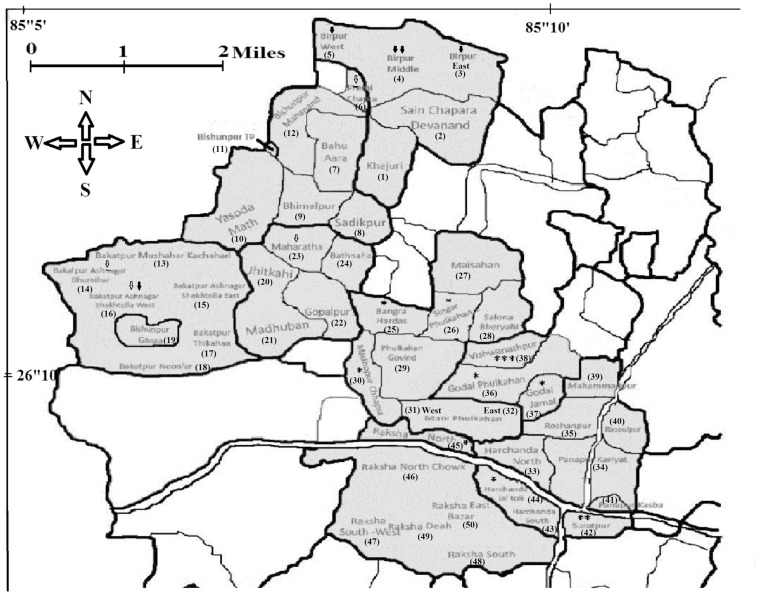
Sand fly collection sites and distribution of infected sand flies in the Kanti block of Muzaffarpur district. Shaded areas represent villages selected for sand fly collection. Distribution of infected sand flies in the summer (hollow arrow), rainy (solid arrow) and winter (star) seasons, respectively.

Sand flies were collected from VL endemic regions of Muzaffarpur district, Bihar, located at 26.07°N 85.45°E that lies in a highly active seismic zone of India. For the sampling, villages were screened on the basis of reported VL cases. Relevant information concerning VL cases was obtained from the Kala Azar Medical Research Centre, Muzaffarpur. Using these data, 50 villages were randomly chosen and 10 houses were selected from each village amounting to a total of 500 houses (Figure 1).

Approval for the study was obtained from the Ethical Committee of the Institute of Medical Sciences, Banaras Hindu University. Written informed consent was obtained from the head of each household.

### Collection of sand flies

To know about the actual status of sand fly infection, collections were conducted during the three different seasons over the course of one year. A total of 500 selected houses were monitored three times during 2010, in the summer (March-April), rainy (August-September) and winter (November-December) seasons. CDC light traps with standard collection cup assemblies (Model 1012 miniature Incandescent Light Trap, John W. Hock, USA) were used for sand fly collection. One light trap was installed inside each house in the evening and left overnight as sand flies are nocturnally active [5], [10]. A total of 10 light traps were installed in 10 houses of each village in one day. In each season, collections were done for 50 days to cover all the villages. Digital thermometers and hygrometers were also installed inside each house to record maximum and minimum temperatures as well as humidity, respectively. On the following day, early in the morning, collected insects were stored in petri dishes containing chloroform coated cotton balls. Sand flies that were resting on the walls of houses were collected using mouth aspirators.

### Morphological identification

Morphological identification was done in two steps. In the first step, sand flies were separated from other insects using an optical microscope while in the second step, sand flies were identified using a stereomicroscope (Carl Zeiss, Singapore) on the basis of morphological characteristics such as the thorax and hairs of abdominal tergites [11]. Collected sand flies belonged to three species: *P. argentipes*, *P. papatasi* and *Sergentomyia babu*. Using morphological characteristics of the anal region, these were further divided into male and female. All the sand flies were stored individually in 70% ethanol at −20°C. Only female *P. argentipes* were selected for this study as they are the only known vector for *L. donovani* infection.

### Sand fly DNA extraction

Each sand fly was individually processed for DNA isolation using a High Pure PCR Template Preparation Kit (Roche, Mannheim, Germany) after crushing the whole sand fly with a sterile polystyrene probe. DNA was quantitated using a NanoDrop 2000 (Thermo Scientific Inc., Wilmington, DE).

### Parasite DNA extraction

*Leishmania* parasite reference strain LEM138 (MHOM/IN/00/DEVI) was cultured in M199 at 26°C and DNA was extracted using DNeasy Blood & Tissue Kit (Qiagen, USA).

Identification of sand fly by PCR-RFLP

**Figure 2:**
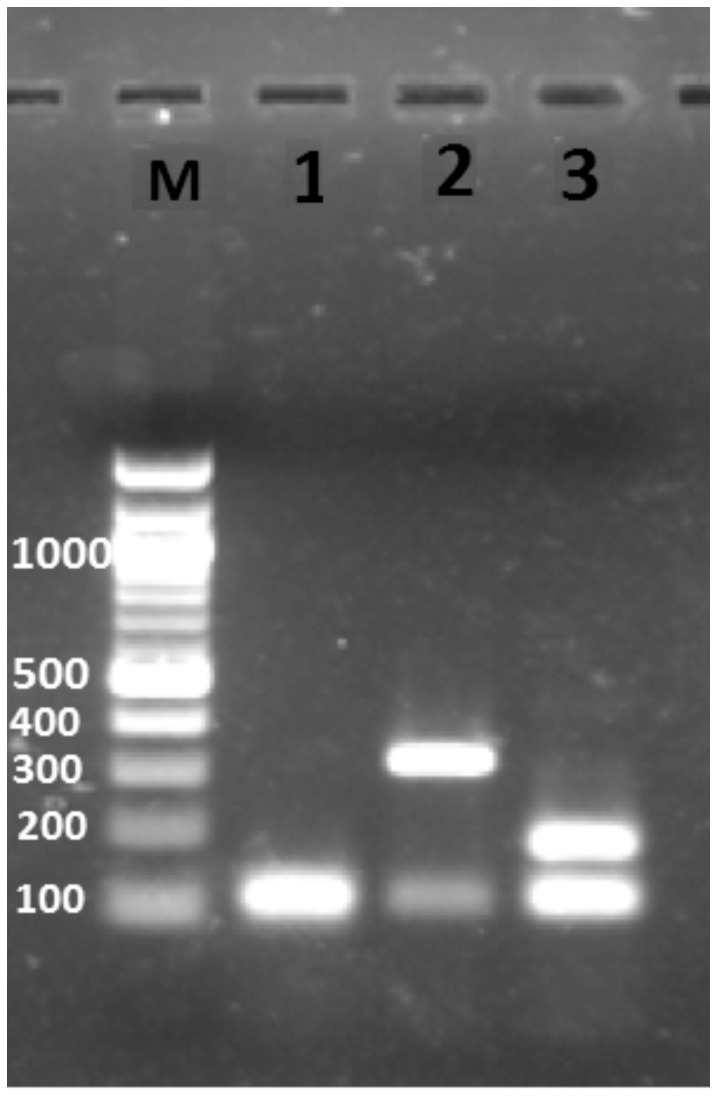
Sand fly species-specific restriction digestion patterns on agarose gel. 18S rRNA gene specific PCR products were digested using the Hinf I restriction enzyme. Lane M: 100 bp marker, Lane 1: *P. argentipes*, Lane 2: *P. papatasi*, Lane 3: *S. babu*.

For molecular confirmation of morphologically identified species using PCR, primers were designed targeting the 18s rRNA coding gene: 5′-TAGTGAAACCGCAAAAGGCTCAG-3′ (forward) and 5′-CTCGGATGTGAGTCCTGTATTGT-3′ (reverse) [12]. The PCR reaction was carried out in a volume of 25 µl using the pair of primers (10 pmol each), 1 U normal *Taq* DNA polymerase (New England Biolabs, United Kingdom) supplemented with MgCl_2_, 10× buffer, 1× BSA, 0.2 mM of dNTPs. For a template, 50 ng of extracted DNA was used while nuclease-free water (QIAGEN, Hilden, Germany) was used as a negative control. PCR conditions were: initial denaturation at 95°C for 5 min, followed by 35 cycles of denaturation at 95°C for 30 sec, annealing at 58°C for 40 sec, extension at 72°C for 30 sec, and a final extension at 72°C for 10 min. Further, restriction digestion was done to discriminate *P. argentipes* from other morphologically similar sand flies. The amplified PCR product was subjected to restriction digestion using Hinf I restriction enzyme (recognition site GATTC; time saver, New England Bio labs, UK) at 37°C for 15 min. The entire reaction product was electrophoresed on a 1.5% (w/v) ethidium bromide (Merck, Darmastaft, Germany) stained agarose gel (Sigma-Aldrich, USA) (Figure 2).

### *Leishmania* specific PCR

**Figure 3:**
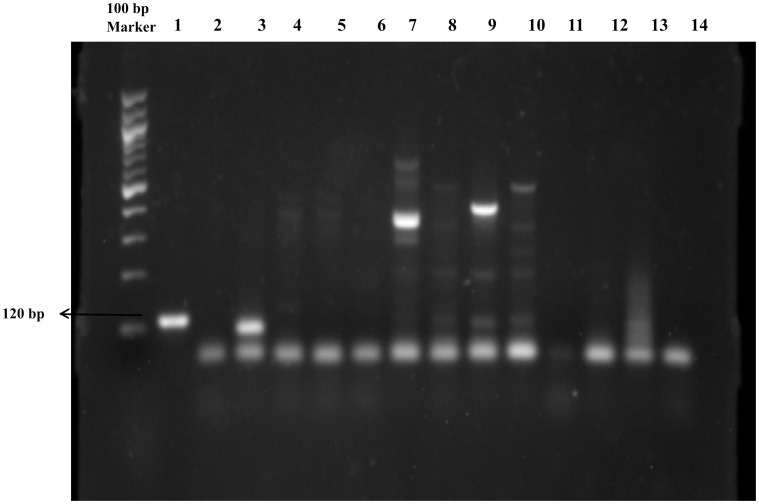
Detection of *L. donovani* in female *P. argentipes* showing amplification of the 116 bp region specific for the minicircle kDNA region. Lane 1: positive control, Lanes 2–13: female sand flies showing amplification in lanes 3, 8, 9 and 10, Lane 14: male sand fly.

Individual *P. argentipes* confirmed by both morphological and molecular methods were used to study the natural infection rates with *Leishmania* using primers specific for the minicircle of the kDNA region of the parasite [13]. The primers used were 5′- CCTATTTTACACCAACCCCCAGT-3′ (forward) and 5′- GGGTAGGGGCGTTCTGCGAAA-3′ (reverse). The DNA of *L. donovani* reference strain, LEM138 (MHOM/IN/00/DEVI), was used as a positive control whereas DNA extracted from male sand flies was used as a negative control. The reaction was carried out in a volume of 25 µL using the primers (10 pM each), 2 U of *Taq* DNA polymerase supplemented with 10× buffer, MgCl_2_, 1× BSA, 25 µM dNTPs and 100 ng of template. The PCR reaction conditions included: initial denaturation at 95°C for 5 min, followed by 40 cycles of denaturation at 95°C for 30 sec, annealing at 58°C for 30 sec, extension at 72°C for 30 sec, and a final extension at 72°C for 10 min. Amplified products were resolved on 1.5% ethidium bromide stained agarose gel (Figure 3).

### Sequencing

Both *P. argentipes* and *L. donovani* specific PCR products were directly used for sequencing. The *P. argentipes* specific PCR product was observed as a single band on the agarose gel, thus leftover primers and dNTPs from PCR products were removed using QIAquick PCR Purification Kit (Qiagen). For *L. donovani* specific PCR reactions, non-specific products were visible, thus the desired product bands were cut and extracted using QIAquick Gel Extraction Kit (Qiagen). Further, the eluted PCR products were sequenced by BigDye Terminator 3.1 cycle sequencing kit (Applied Biosystems, Foster City, CA, USA) and run through an ABI 3133 sequencer.

### Quantitative real-time PCR

**Figure 4:**
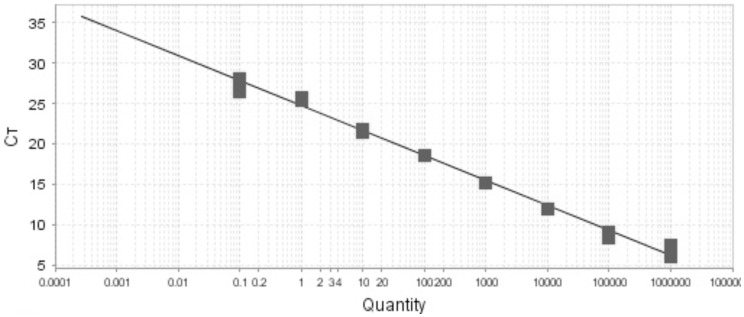
Standard curve obtained by plotting the Ct against serial 10-fold dilutions of *L*. *donovani* DNA, starting at 10^6^ parasites per reaction. Ct represents the fractional cycle number reflecting a positive PCR result differentiated from background.

*Leishmania* specific minicircle kDNA primers as described above were used to quantitate parasite load in the PCR-positive sand flies [13]. Determination of parasite load was done using a standard curve made with 10-fold serial dilutions of *Leishmania* DNA spiked with a fixed amount of sand fly DNA. Parasites were counted using a Neubauer chamber and DNA was eluted with final concentration of 2.5×10^5^ parasites/µl which was diluted 8 times and a standard curve was plotted. A standard curve was obtained by plotting the Ct against the known target quantity on a common log scale (Figure 4). Parasitic load in the infected sand flies was calculated by comparing Ct values with the standard plot (Table 1). Along with the PCR positive samples, PCR negative samples, male sand fly samples and no template master mix were used as negative controls on each plate.

**Table 1:** Parasitic load in *Leishmania*-infected sand flies.

Season	No. of infected sand flies	No. of parasites in infected sand flies		
		Unfed	Fed	Gravid
Summer	4	17		1, 6, 6987
Rainy	5	1, 2, 10		1, 253
Winter	12	1, 1, 2, 2, 4, 6, 13, 1294	1, 1, 1, 2	

For the *P. argentipes* specific SYBR Green assay, the target was the 18S rRNA encoding gene of the sand fly (GenBank accession number AJ244360.1). For amplification, primers were designed as 5′-TGTGTGCGTTCACTGTCAAAGGTG-3′ (forward) and 5′- AAGTAACTGTACCGGCCCACA-3′ (reverse) to amplify a 188-bp fragment to check for inter-sample variation in sand fly DNA amplification.

The parasitic load of all *Leishmania* specific PCR-positive sand flies as well as 150 PCR-negative sand flies (50 from each season randomly selected) was calculated. Q-PCR reactions were set up with 10 µl reaction mixture containing 5 µl of 2× SYBR Green reagent (Applied Biosystems), 5 pM each of forward and reverse primers, and 100 ng of each DNA sample. Reactions were analyzed in MicroAmp optical 96 well reaction plates (Applied Biosystems) using a 7500 Real-Time PCR System (Applied Biosystems). The thermal cycling conditions were similar for both *L. donovani* and *P. argentipes* specific Q-PCR: initial denaturation stage at 95°C for 8 min, amplification stage (40 cycles at 95°C for 15 sec and 60°C for 1 min each), melting stage (95°C for 15 sec, 60°C for 1 min and 95°C for 30 sec) followed by cooling at 60°C for 15 sec. Each sample was tested in duplicate.

### Data analysis and statistical methods

**Figure 5:**
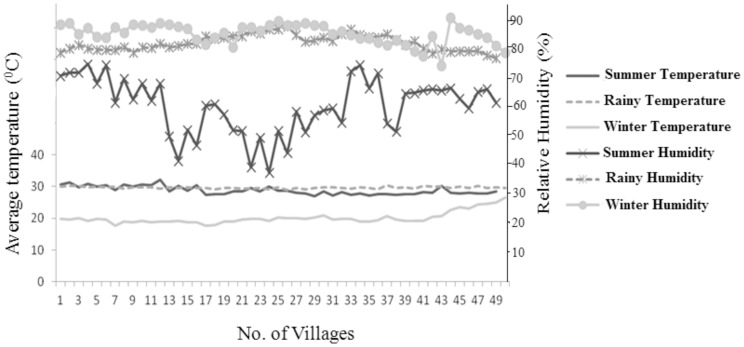
Variation in average temperature and relative humidity of all 50 villages included in the study. Average temperature (Celsius) and relative humidity (%) shown for the summer, rainy and winter seasons.

Temperature and humidity were measured in each house involved in the study. A graph was plotted indicating average temperature and relative humidity corresponding to all the 50 villages for each season (Figure 5). There was marked variation in average temperature and relative humidity between seasons.

For Q-PCR, generation of amplification plots, mean values, standard curve analysis and calculation of the melting temperature of each amplicon were done directly using the 7500 software v2.0.1 (Applied Biosystems). The fluorescence threshold limit of the ABI 7500 Real-Time PCR System was set at 0.02 as recommended by the manufacturer.

## Results

### Collection of female *P. argentipes*

**Figure 6:**
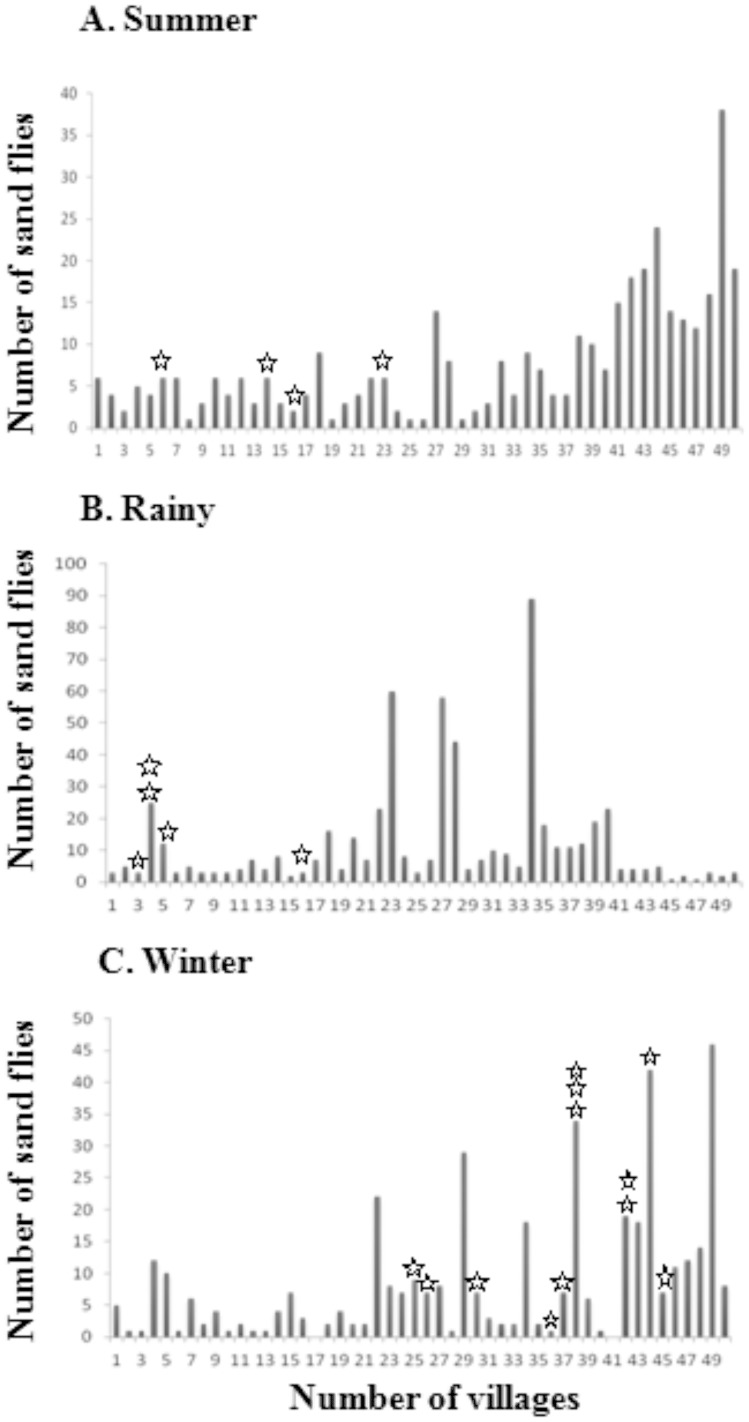
Variation in total collected and infected sand flies from all 50 villages in each season. Total collected sand flies (solid hologram) and infected sand flies (star shaped) from each village, shown for the **(A)** summer, **(B)** rainy and **(C)** winter seasons.

The total collection of sand flies varied according to season: the number of female *P. argentipes* were 384 (blood fed 58, unfed 172, gravid 154), 591 (blood fed 59, unfed 378, gravid 154) and 422 (blood fed 54, unfed 208, gravid 160) in th esummer, rainy and winter seasons, respectively. Village-wise variation in the number of collected sand flies is shown in Figure 6. All of the morphologically identified *P. argentipes* were validated using species-specific PCR-RFLP. Sixty randomly chosen *P. argentipes* specific PCR products (20 from each season) were sequenced using primers specific to the sand fly 18S rRNA coding gene. Sequence analysis confirmed that all of the samples were similar to known *P. argentipes* 18s rRNA sequences (Genbank Accession number JX910368).

### *L. donovani* infection in sand flies

After amplification of the *Leishmania*-specific minicircle kDNA region, a 116 bp product was observed in the infected female *P. argentipes* samples. The amplified PCR products were eluted from the agarose gel and sequenced using the same primers. Sequence analysis confirmed that all of the amplified bands had 100% homology with *L. donovani* minicircle kDNA.

Using *L. donovani* specific PCR, seasonalal variation in the infection level of sand flies was observed. The positivity rate for the entire year was 1.50% (21/1397). However, the infection rates were 1.04% (4/384) in summer, 0.85% (5/591) in the rainy season, and 2.84% (12/422) in winter. Female sand flies belonged to three categories – blood fed, unfed and gravid – that showed variation in rates of infection. Amongst the infected sand flies, the highest rate of infection was observed in the blood fed group at 2.34% (4/171), followed by the unfed group at 1.58% (12/758), and the gravid group at 1.07% (5/468). The village-wise distribution of infected sand flies shown in Figure 1 and Figure 6.

### Parasitic loads of infected sand flies

Parasitic load was investigated by real-time PCR using the standard curve absolute quantification method to estimate the number of *Leishmania* parasites in naturally-infected sand flies. Parasitic loads of infected sand flies are shown in Table 1. Parasitic loads were highest in gravid sand flies, followed by unfed and blood fed ones.

## Discussion

Although VL is considered a significant public health problem in the Indian subcontinent, very little research has been done on the behaviour of sand fly vectors. Correct identification of the vector population and the rate of *Leishmania* infection in endemic areas is fundamental for understanding the epidemiology of the disease that may help inform vector control programmes.

PCR based methods were used for the study as we needed a highly robust method to include a large sample size. For sand fly identification, all the collected insects were first morphologically identified and differentiated in various groups. For the present study, only female *P*. *argentipes* were selected as sexual differentiation is not possible by molecular methods. Further PCR and restriction digestion were done to confirm the sand fly species. Using molecular methods, various sand fly species that co-exist in same area can be easily differentiated. In endemic areas of Muzaffarpur, there are three prevalent species of sand fly: *P*. *argentipes*, *P. papatasi* and *S. babu*. With restriction digestion, each species displayed an easily identifiable specific band pattern on agarose gel.

Morphological identification of *Leishmania* parasites by microscopy in sand flies collected in the field may take a long time, as parasite load in naturally infected sand flies could be very low [14]. For highly sensitive *Leishmania* parasite detection, the minicircle kDNA region was selected as it is present as approximately 10,000 copies per parasite [15]. Two sets of primers were used for the identification of *P. argentipes* and *L. donovani*, respectively. Detection of parasite infections in the insects using morphological methods can be time consuming, as consistent observation under a microscope is needed for large sample sizes and samples cannot be used further as there may be a risk of contamination after dissection. PCR based methods targeting various conserved genes such as minicircle kDNA [16], [17], ITS [18], [19], Cytochrome b gene [20] and 18S rRNA [21] encoding regions have been used for detection of the *Leishmania* parasite in sand fly vectors. As previously reported, since the parasitic load in naturally-infected sand flies is less, a highly sensitive primer was needed. PCR is preferred over microscopy as a large sample size can be processed simultaneously and it is more sensitive for detecting infection. Also, PCR is time-saving and has been used for similar studies in other countries, allowing for comparability of results [20], [22].

In studies of the prevalence of *Leishmania* infection in sand flies, collection and processing is usually done by two methods, either by pooling sand flies or by testing them individually. The pooling method is easier, time-saving and efficient [21], [22], [23], [24]. However, the exact number of infected sand flies in a positive pool is impossible to calculate and information on individual sand flies, such as parasitic load or blood meal host, can be lost. Such drawbacks can be overcome by assaying individual sand flies. Previous studies have shown prevalences of *Leishmania* infection in natural sand fly populations ranging from 0.7 to 2.0% [19], [25], [26], [27]. Considering the limitations inherent in pooling samples, as well as the low expected infection rates, each sand fly was processed individually to determine the exact infection rate in our study.

Based on the seasonal collection of sand flies throughout the year, we found that sand flies are highly prevalent in rainy season. The aim of the present study was to describe the seasonal variation in *L. donovani* infection in the vector population. The overall percentage of *L. donovani* infection in female *P. argentipes* was 1.5% although this varied by season. The reason that the lowest rate of infection was observed in the rainy season might be due to the flushing effect of rainfall on immature, emerging sand flies. With the drainage of water during winters, immature sand flies start developing on the ground and thus during winter, more numbers of mature and potentially infective sand flies exist [28]. The highest parasitic load in summer might be due to the occurrence of the highest number of gravid sand flies as they need to ingest a greater quantity of blood for their eggs to develop. At the same time, there are more active VL patients during summer, so the flies may ingest a higher number of parasites while taking a blood meal from an infected human host.

This observation may be beneficial for understanding VL dynamics as behavioural changes in vectors can have direct impact on disease transmission. Vector control is still the most powerful tool for preventing vector borne diseases. Thus, knowledge of the infection status of the vector population has proved crucial for disease control programmes. With the present study, maximum infection was observed in sand flies collected in the months of November-December with 2.84% infection; consequently, the infectivity of sand flies should be maximum during these months. The time interval between the bite of an infected sand fly and development of disease is between 2 to 6 months. This finding is supported by another study in which monthly variations in the incidence of VL were observed, with the highest incidence occurring in March-April [9]. The present study may be beneficial for vector control programmes in targeting the most effective period for insecticidal spraying.
